# Activating Hydrogen Evolution Reaction on Carbon Nanotube via Aryl Functionalisation: The Role of Hybrid sp^2^–sp^3^ Interface and Curvature

**DOI:** 10.3390/nano13142122

**Published:** 2023-07-21

**Authors:** Muhammad Ahmed, Gurpreet Kour, Ziqi Sun, Aijun Du, Xin Mao

**Affiliations:** 1 School of Chemistry and Physics, Faculty of Science, Queensland University of Technology, Gardens Point Campus, Brisbane, QLD 4001, Australia; 2 QUT Centre for Materials Science, Queensland University of Technology, Gardens Point Campus, Brisbane, QLD 4001, Australia

**Keywords:** aryl-L (L = Br, CCH, Cl, CO_2_CH_3_, F, I, NO_2_, t-butyl), hydrogen evolution reaction, sp2–sp3 hybrid interface, hydrogen-binding Gibbs free energy, electrocatalyst, density functional theory

## Abstract

The hydrogen evolution reaction (HER) is a remarkable mechanism which yields the production of hydrogen through a process of water electrolysis. However, the evolution of hydrogen requires highly conductive and stable catalysts, such as the noble metal platinum (Pt). However, the problem lies in the limitations that this catalyst and others of its kind present. Due to limited availability, as well as the costs involved in acquiring such catalysts, researchers are challenged to manufacture catalysts that do not present these limitations. Carbon nanotubes (CNTs), which are nanomaterials, are known to have a wide range of applications. However, specifically, the pristine carbon nanotube is not suitable for the HER due to the binding free energy of its positive H-atoms. Hence, for the first time, we demonstrated the use of the proposed aryl-functionalised catalysts, i.e., Aryl-L@SWCNT (L = Br, CCH, Cl, CO_2_CH_3_, F, I, NO_2_, or t-butyl), along with the effect of the sp2–sp3 hybridised interface through the density functional theory (DFT). We performed calculations of single-walled carbon nanotubes with multiple aryl functional groups. By employing the DFT calculations, we proved that the curvature of the nanotubes along with the proposed aryl-functionalised catalysts had a noteworthy effect on the performance of the HER. Our study opens the door to investigating a promising group of catalysts for sustainable hydrogen production.

## 1. Introduction

Society is overburdened with the day-to-day worsening of living and health conditions. Over the years, with the growth of the world’s population, there has also been an incredible increase in the demand for energy. There are, however, many problems with the current energy infrastructure, such as the emission of CO_2_, acid rain, and the greenhouse effect. A main concern for current researchers is to establish cost-efficient, clean energy production [1,2]. Hydrogen is recognised as the most efficient and sustainable clean energy carrier and an alternative to the hydrocarbon in fuel cells due to its eco-friendliness and high energy density. In fact, hydrogen is the most abundant and lightest element. Further, hydrogen is also easily accessible. Hydrogen can be produced from water, which is abundant in nature, can be transported over long distances, and can also be stored in various ways. In comparison to other fuels, hydrogen is rich in energy per unit of mass [2,3].

Evolving hydrogen from water splitting using energy generated from renewable resources, such as wind or solar power, is the most sustainable method for hydrogen production [4,5]. The hydrogen evolution reaction (HER) is a well-documented reaction, which yields the production of hydrogen at the cathode level and oxygen at the anode level. The reduction in energy consumption is reliant on two parameters: the current density and the overpotential [6,7,8,9]. Particularly, the HER becomes impractical due to its high overpotential [10,11].

When considering the process of the HER, the first step in the mechanism begins with the adsorption of hydrogen, which is referred to as the Volmer reaction. The next step depends on the nature of electrocatalysis and can either proceed with two adsorbed hydrogens, which is referred to as the Tafel reaction, or proceed with an adsorbed H with a H^+^ from the electrolyte, which is referred to as the Heyrovsky reaction. Both of these pathways yield a H_2_ molecule [12]. Therefore, in order to facilitate the adsorption process, an electrocatalyst is required. The electrocatalyst must be stable in nature and have a strong conductivity, such as the noble metal Pt. Pt is proven to be one of the best catalysts for the HER due to its high exchange current density and smaller Tafel slope at approximately 30 mV dec^−1^. In addition, the hydrogen-binding Gibbs free energy (∆G_H_) on Pt is close to zero (∆G_H_ ≈ 0). However, due to the high cost and the scarcity of Pt and its counterparts, such as ruthenium (Ru) or palladium (Pd), their commercial use is practically impossible. In turn, it is difficult to achieve sustainable hydrogen production [12,13]. Therefore, researchers are challenged to manufacture catalysts capable of exceeding these limitations [14]. To date, research has been performed with transition metals or even alloys, but these could not outperform the benchmark catalysts for the HER [15,16]. As a result, for future water splitting, non-precious or non-metallic catalysts with high HER activity, compatibility, and stability are widely desired [17,18].

Moreover, carbon nanotubes have sparked an interest for their potential use as non-noble metal-free catalysts [19]. In fact, a distinction has been made between single-walled carbon nanotubes (SWCNTs) and multi-walled carbon nanotubes (MWCNTs). SWCNTs have attracted much more attention in many lines of research, including catalysis. SWCNTs are consistently composed of one-layer atoms, while MWCNTs consist of many layers of graphitic carbon [20]. Moreover, SWCNTs are divided into three types, including the armchair, zigzag, and chiral. This has prompted further studies on MWCNTs, more than on SWCNTs [21].

Furthermore, it is often argued that using pure carbon nanotubes (p-CNTs) would be favourable for processes that need electron transfer stages, since the electronic characteristics of these CNTs are intact and may be fully utilised [22]. CNTs do most often require modification with an organic functional group. However, p-CNTs have been reported to be active in several reactions. In fact, the studies in this line of research are increasing. Although attempts have been made to develop catalytic processes using as-produced carbon nanotubes, the number of reports is far lower than that for modified carbon nanotubes [22,23,24]. Carbon nanotubes have evolved as distinct carbon allotropes with promise for use in catalysis. Their primary use is providing support for inorganic metal catalysts, such as molecular catalysts, metal oxides, metal nanoparticles, and more complicated hierarchical hybrids. Several studies have demonstrated that they may operate as metal-free catalysts, with performance typically exceeding that of other carbon compounds [25,26,27,28,29,30,31].

Furthermore, in recent years, several non-noble metals, e.g., graphene-covered non-noble metal catalysts and MXenes, have been explored as potential HER catalysts [32,33,34,35,36]. In fact, MXenes are environmentally friendly, and have superior biocompatibility. The scientific community has been astonished as to the wide range of potentiality for energy conversion and storage that is observed in relation to MXenes. However, the limitations in utilizing these for graphene support and surface functionalisation affect their practicality, along with the restricted active sites on the edges [8,32,33,34,35,36]. On the other hand, we have carbon with its natural abundance, adaptive molecular structure, strong conductivity, and high tolerance to alkaline/acidic environments. In fact, studies on altering the environment have been conducted, as most of the electrocatalysts work well in an acid medium, in addition to an alkaline medium [37,38,39,40,41,42,43]. This is one reason carbon-based materials are extensively employed in the development of many types of catalysts for energy conversion. Further, studies with carbon materials have already shown a promising future for the oxygen reduction reaction (ORR). Moreover, carbon can occur in various forms of allotropic structures with low dimensions, such as 2D graphene [44,45,46,47,48], 1D carbon nanotubes [49,50,51], and 0D fullerenes [52,53,54]. All of these different forms of carbon have been extensively explored for the development of green and renewable energy technologies, such as lithium-ion batteries as well as solar and fuel cells [55,56,57,58,59,60,61]. Given its ever-increasing relevance in society, the energy industry has emerged as one of the most investigated domains in recent times. Therefore, in this paper, we explore the use of non-precious aryl-functionalised (aryl-L, L = Br, CCH, Cl, CO_2_CH_3_, F, I, NO_2_, and t-butyl) catalysts for water splitting under standard conditions.

To date, work with aryl-functionalised single-walled carbon nanotubes has been performed with electroluminescence, but the number of studies remains far too low. In studies conducted in this emerging area of research, the SWCNT display exemplary optical and electronic properties [62]. However, progress is limited due to the poor luminescence of the SWCNT films. In turn, researchers have introduced sp^3^ defects, which result in a red-shifted photoluminescence with a prolonged lifetime and a higher photoluminescence. These types of aryl-decorated nanotubes are feasible to synthesise in a laboratory setting and similar types have already been synthesised [62,63,64,65,66].

In this research, we have performed the calculations of single-walled carbon nanotubes with multiple aryl functional groups, i.e., Aryl-L@SWCNT (L = Br, CCH, Cl, CO_2_CH_3_, F, I, NO_2_, t-butyl), as a new electrocatalytic for the HER catalysts due to their electrical characteristics [67]. Our proposed aryl-functionalised catalyst is simply composed of non-metal atoms attached to this carbon nanotube structure, and its performance as a novel HER catalyst is explored in depth in this paper. The binding Gibbs free energy of the H-atom on the Aryl-L@SWCNT catalysts is extremely close to zero, which is similar to or better than that for other well-studied HER catalysts, including the nanostructured MoS_2_ materials and the state-of-the-art Pt catalysts. According to our research, the electron transfer from the aryl functional group to a carbon nanotube significantly impacts the HER. This impact was studied through the use of DFT calculations. Our findings highlight a novel HER catalyst that can serve as an alternative to noble metal catalysts and may be used in future scientific studies.

## 2. Computational Details

First-principle DFT calculations were performed to better understand the function of aryl groups at the sp^2^–sp^3^ carbon interface. In comparison to experimental testing, first principles calculations can be performed in any degree, thereby making it possible to determine the makeup or the energy use. Further, with the use of such calculations, we can control the inputs, which eliminates the possibility of unknown variables. However, it must be noted that while this will help us in understanding the theoretical framework, the actual modelling of the electrocatalytic reaction is complex. In our research, the theoretical overpotential was projected using Nørskov and co-workers’ computational approach [68]. We used the DFT as demonstrated in the Vienna Ab-initio Simulation Package (VASP) code to perform the calculations [69,70,71]. To study nuclei and core electrons, the frozen-core projector augmented wave (PAW) method [72] was used, and to describe the exchange–correlation interactions, the generalised gradient approximation [73] in the form of the Perdew–Burke–Ernzerhof functional [74] was used. Spin polarisation was also taken into account for all calculations. The energy level of plane waves was capped at 500 eV, while the force and energy were converged at the optimum level of 0.001 eV/Å and 10^6^ eV, respectively. The pH was set to 0 with no bias potential during the calculation.

The following equation describes the overall HER pathway:

(1) H++e−→12H2

The reaction occurs at an electrode and forms an intermediate state:

(2) $$H^{+}+e^{-}{+}^{*}\to H^{*}$$

(3) $${2H}^{*}\to H_{2}$$

where (*) represents a free site on the surface and H* represents a hydrogen atom absorbed on the surface.

The final hydrogen evolution step may also be written as:

(4) $$H^{+}+e^{-}+H^{*}\to H_{2}$$

The free energy of the adsorption of the atomic hydrogen (∆G^0^_H*_) is calculated by the equation below:

(5) $$\Delta G_{H^{*}}^{0}=\Delta E_{H}+\Delta E_{ZPE}-T\Delta S_{H}$$

In Equation (5), ∆E_H_ provides the differential hydrogen adsorption energy and can be written as:

(6) ΔE=EH*−E*−12EH2

where the asterisk (*) denotes the catalyst. E_H*_ represents the total energy of the catalyst plus one hydrogen (H)-adsorbed atom; $E_{*}$ represents the total energy of the catalyst without an adsorbed hydrogen (H) atom; and EH_2_ represents the energy of the hydrogen gas (H_2_). The difference corresponding to the zero-point energy between the gas phase and the adsorbed state is represented by ∆E_ZPE_. The contributions of catalysts to both ∆_ZPE_ and ∆S_H_ are minuscule; and therefore, they can be neglected. The ∆E_ZPE_ is calculated using the given equation [75]:

(7) ΔEZPE=EZPEH−12EZPEH2

where E^H^_ZPE_ is the zero-point energy of one adsorbed atomic hydrogen on the catalyst without the effect of the catalyst; $E_{ZPE}^{H_{2}}$ is the zero-point energy of H_2_ in the gaseous phase; S^0^_H2_ is the entropy of H_2_ gas at the standard condition [76].

∆S_H_ can be calculated as:

(8) ΔSH≅−12SH20

Based on this, the calculated vibrational frequency for H_2_ gas is 4390 cm^−1^, while the vibrational frequency of H adsorbed on aryl-L is 2810 cm^−1^. Since this frequency is not sensitive to the aryl atom, overall corrections are made as:

(9) $$\Delta G_{H^{*}}^{0}=\Delta E_{H}+0.24eV$$

In the volcano-shaped diagram, the average Gibbs free energy of hydrogen adsorption (∆G^0^_H*_) on catalysts is used to calculate the theoretical exchange current, i_0_. Using Nørskov’s assumption [77], if ∆G^0^_H*_ = ≤ 0, the following expression for the exchange current at pH = 0 is applicable:

(10) $$i_{0}=-ek_{0}\frac{1}{1+\exp\left(-\frac{\Delta G_{H^{*}}^{0}}{k_{b}T}\right)}$$

If the ∆G^0^_H*_ > 0, the exchange current is calculated using:

(11) $$i_{0}=-ek_{0}\frac{1}{1+\exp\left(\frac{\Delta G_{H^{*}}^{0}}{k_{b}T}\right)}$$

## 3. Results and Discussion

**Figure 1:**
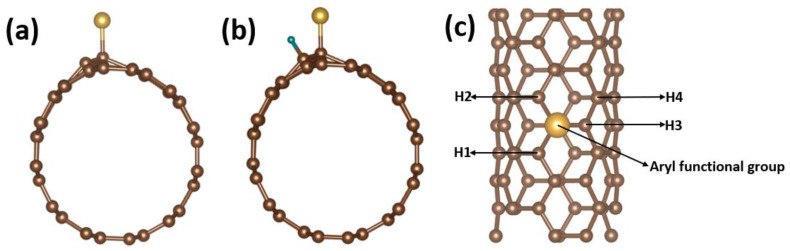
(**a**) Model for SWCNT on which an aryl-L (L = Br, CCH, Cl, CO_2_CH_3_, F, I, NO_2_, or t-butyl) functional group was attached. (**b**) Model for SWCNT on which an aryl-L (L = Br, CCH, Cl, CO_2_CH_3_, F, I, NO_2_, or t-butyl) functional group was attached and an H was adsorbed. (**c**) Model for SWCNT on which an aryl-L (L = Br, CCH, Cl, CO_2_CH_3_, F, I, NO_2_, or t-butyl) functional group was attached, representing different sites (H1, H2, H3, and H4) on which H was adsorbed. Colour code: brown, C; golden, aryl-L; green, H.

The aryl-L structures were created by adding one aryl functional group to the carbon nanotube, as demonstrated in Figure 1 below and Figure S5 in the Supplementary Materials. Previously, a set of aryl functional groups were attached in an experimental setting. The adsorption shape was then improved by adding H-atoms to various places on Aryl-L@SWCNT (see Figures S6–S9 in the Supplementary Materials). The adsorption of H-atoms on top of surface C-atoms is the most energetically stable location. The binding free energy (∆G_H_) of H-atoms is computed as a suitable descriptor for HER activity evaluation [78]. Strong adsorption on the catalyst is indicated by a reduced free energy (∆G_H_), while a weak H-binding is indicated by a higher free energy (∆G_H_). When the free energy (∆G_H_) is near zero, the best HER activity is produced. The hydrogen-binding Gibbs free energy (∆G_H_) of a pristine carbon nanotube (SWCNT) is too positive (∆G_H_ = 1.04 eV), relative to the ideal value of ∆G_H_, indicating a weak interaction between adsorbed H and SWCNT, resulting in poor HER reaction kinetics. The Aryl-L@SWCNT, on the other hand, displayed a dramatically increased activity for HER when aryl functional groups (Aryl-L) were attached to the carbon nanotube (SWCNT). Br, CCH, Cl, CO_2_CH_3_, F, I, NO_2_, and t-butyl are among the aryl functional groups attached to the carbon nanotube, and the free energy *(*∆G_H_*)* of each species is computed.

**Figure 2:**
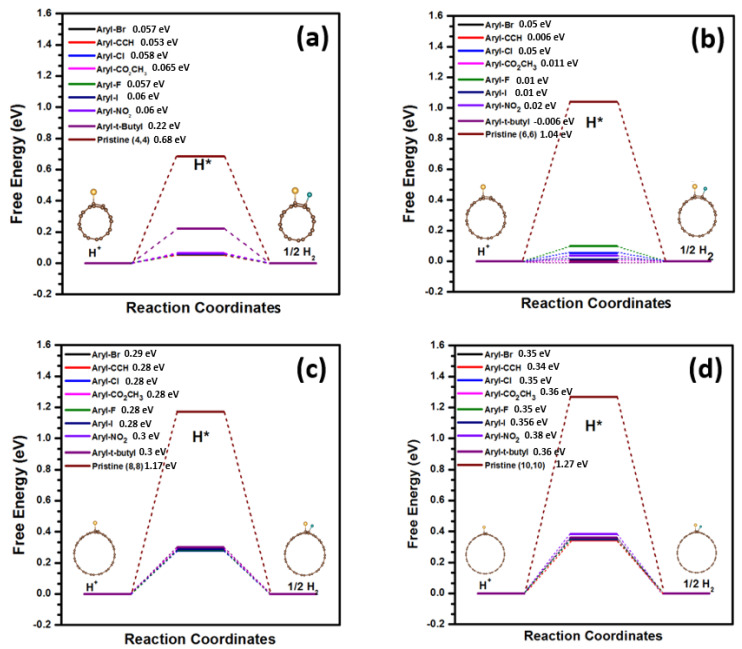
(**a**) The calculated free-energy diagram of the hydrogen evolution reaction, U = 0 V, under standard conditions for armchair (4,4) Aryl-L@SWCNT catalysts. (**b**) For armchair (6,6) Aryl-L@SWCNT catalysts. (**c**) For armchair (8,8) Aryl-L@SWCNT catalysts. (**d**) For armchair (10,10) Aryl-L@SWCNT catalysts. L = Br, CCH, Cl, CO_2_CH_3_, F, I, NO_2_, or t-butyl. The H adsorption active site was H1.

In our research, we studied four armchair nanotubes, (4,4), (6,6), (8,8), and (10,10), each having different curvatures under standard conditions, to examine the influence of the sp2–sp3 carbon interface on the catalytic activity of HER (Figures S1–S4 in the supplementary Materials show the H adsorption free energies for all possible active sites). Among all four types of Aryl-L@SWCNT, (L = Br, CCH, Cl, CO_2_CH_3_, F, I, NO_2_, and t-butyl), we demonstrated that the lowest free energy (∆G_H_) is presented by the armchair (6,6) (see Figure 2), which exhibited excellent HER performance. Other aryl catalysts (armchair (4,4), (8,8), and (10,10)) with extremely high or extremely low Gibbs free energies for atomic hydrogen adsorption are also included in Figure 2 for comparative study. The value of free energy for the pristine carbon nanotube (p-SWCNT) is very unstable (∆G_H_ = 1.04 eV), as illustrated in Figure 2. For the hybridised sp2–sp3 carbon interface, the hydrogen-binding Gibbs free energy of Aryl-L@SWCNT (L = Br, CCH, Cl, CO_2_CH_3_, F, I, NO_2_, or t-butyl) catalysts can be greatly decreased to near zero “0” (−0.1 eV < ∆G_H_ < H 0.1 eV). The HER performance is equivalent to or better than that of the current generation of Pt (∆G_H_ = −0.09) catalyst and is exceedingly superior to that of the MoS_2_ (∆G_H_ = 0.13) catalyst [33,34,77,79].

**Figure 3:**
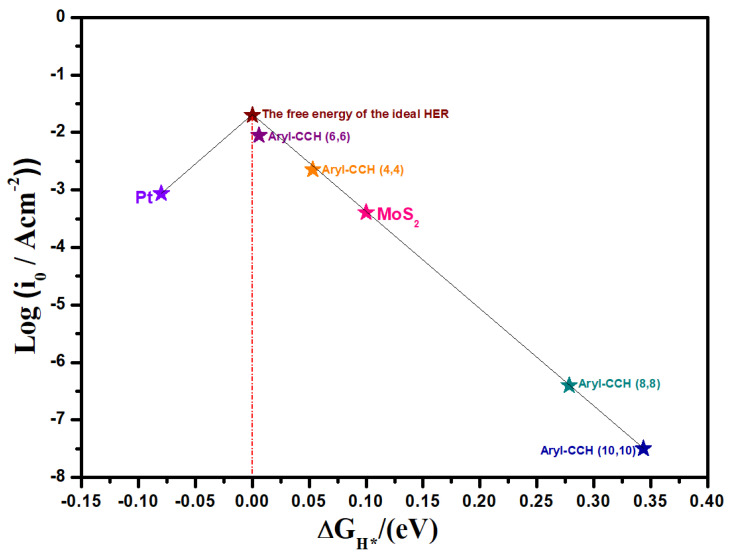
Volcano curve of exchange current (i_0_) as a function of the average Gibbs free energy of hydrogen adsorption (∆G) for various aryl functional groups attached on the carbon nanotube. Values closer to the peak represent stronger catalytic activity.

To compare the HER performance of Aryl-L@SWCNT, we plotted a volcano curve for the best value in each of the four nanotubes displayed in Figure 3 (including pristine C60). Based on Equations (7) and (8), the Gibbs free energies of hydrogen adsorption (∆G_H_) on Aryl-L@SWCNT (H adsorption active site was H1) are utilised to calculate the theoretical exchange current (i_0_). The i_0_ position and ∆G_H_ values of Aryl-L@SWCNT may be used to assess its HER performance relative to the volcanic peak (a location closer to the peak implies a stronger catalytic activity) [77]. Aryl-L catalysts with negative and positive ∆G_H_ values are located at the left and right legs of the volcano curve, respectively. Near the apex of the volcanic curve are catalysts with ∆G_H_ values close to zero. On the other hand, Aryl-L@SWCNT (L = Br, CCH, Cl, CO_2_CH_3_, F, I, NO_2_, or t-butyl) catalysts for armchair ((8,8) and (10,10)) have either too negative or too positive ∆G_H_ values, making them unsuitable for releasing or adsorbing hydrogen during the hydrogen evolution process. The armchair (6,6) Aryl-CCH@SWCNT catalyst is an excellent candidate for HER, with ∆G_H_ values near zero “0”. When comparing the results of the other two (8,8) and (10,10) nanotube Aryl-L@SWCNT catalysts to those of other (4,4) and (6,6) Aryl-L@SWCNT catalysts, there is a considerable off-centre shift for (8,8) and (10,10) Aryl-L@SWCNT. This may be due to the extra-small curvature of the nanotube (8,8) and (10,10) Aryl-L@SWCNT catalysts, which results in a stronger bonding energy of the hydrogen species. In other words, small curvatures lead to weak adsorption, while large curvatures lead to strong adsorption. Therefore, the results reflect a binding free energy away from zero and low exchange current density (i_0_).

**Figure 4:**
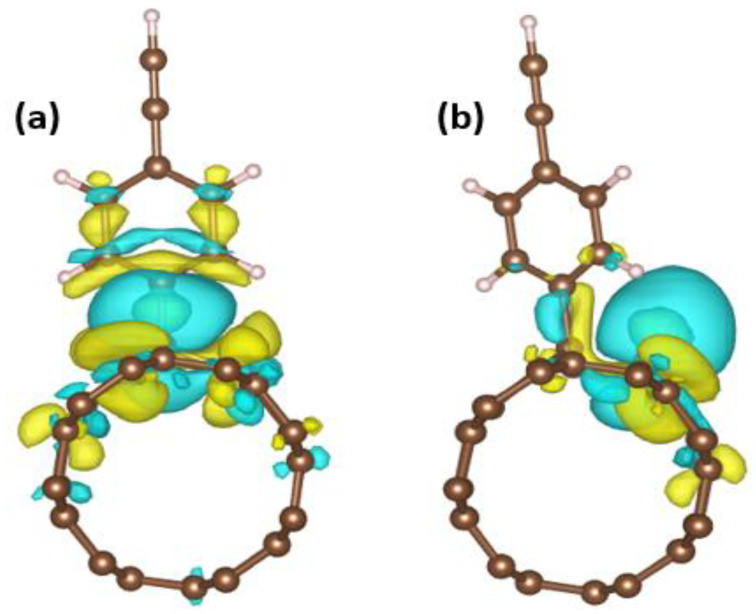
Charge density difference (∆ρ(r)) plots for (**a**) Aryl-CCH@SWCNT and (**b**) Aryl-CCH@SWCNT-H. Yellow and cyan iso-surfaces represent electron accumulation and electron depletion; the iso-surface value for (**a**) is 0.000752631 eÅ^−3^, and that for (**b**) 0.000954316 eÅ^−3^.

The attached aryl functional groups have a considerable influence on the adsorption of atomic hydrogen on the carbon nanotube, as discussed above. This is due to the carbon nanotube’s electron-acceptor properties, which allow it to quickly receive electron density from the H-atom within the nanotube. To further understand this effect, we use Aryl-CCH@SWCNT as an example and use the following equation to determine the charge density difference (**∆**ρ(r)) to analyse the distinction between the system’s charge density and the reference charge densities of the system’s constituent structures. This recognises the depletion and accumulation of charges and depicts charge redistribution caused by interacting materials, for three typical systems, Aryl-CCH@SWCNT, Aryl-CCH@SWCNT-H, and SWCNT, based on the given equation:

**∆**ρ(r) = ρ(r)_Aryl-CCH@SWCNT-H_ − ρ(r)_SWCNT_ − ρ(r)_Aryl-CCH_ (12)

where ρ(r)_Aryl-CCH@SWCNT-H_ indicates the charge density of the system Aryl-CCH@SWCNT with an adsorbed H-atom; ρ(r)_SWCNT_ represents the charge density of the system SWCNT without an adsorbed H-atom; and ρ(r)_Aryl-CCH_ is the charge density of the Aryl-CCH estimated for the atoms of H and aryl-CCH at the same coordinates as those in the Aryl-CCH@SWCNT-H and Aryl-CCH@SWCNT systems, respectively. Figure 4 shows the result of the charge density difference.

In the Bader charge analysis, we found that, after the adsorption of the H-atom on the active site, the aryl-CCH has the highest charge compared to the other aryls (Table S1 in Supplementary Materials). The implanted aryl-CCH group causes a rise in the electronic charge density around the carbon nanotube surface, which is surrounded by the aryl-functionalised group, as seen in Figure 4a. The yellow iso-surface represents the electron accumulation, while the cyan iso-surface represents the electron depletion. There is a considerable charge density redistribution in the presence of aryl-CCH, potentially because of the attached aryl-CCH group carrying a very effective charge transfer throughout the entire structure when the H-atom is adsorbed. The dopant atom (H-atom) clearly ionises the carbon nanotube, resulting in a significant H adsorption on Aryl-L@SWCNT and an increased HER activity. Moreover, we further analyzed six of the newly developed aryl catalysts to examine the effects of the solvated surface with water solvent. The findings revealed that solvation induced a minor alteration in the Gibb’s free energy such as CCH without solvation shows 0.005888 eV but with solvation showed 0.011488 eV (Table S2 in Supplementary Materials). Lastly, we ran some additional calculations to explore how the hydrogen (H) coverage impact the calculated free energies. Figure S10 in the supplementary materials, shows the results obtained, further enhancing the understanding of the complex interplay between these factors in the catalytic process.

## 4. Conclusions

As stated, the amount of research conducted with aryl-functionalised single-walled carbon nanotubes is far too low. In fact, the only studies with aryl-functionalised single-walled carbon nanotubes have been performed using electroluminescence. These types of aryl-decorated nanotubes are feasible to synthesise in a laboratory setting. However, in our research, DFT simulations were used to study the HER activity of the carbon nanotube and a variety of aryl-L (L = Br, CCH, Cl, CO_2_CH_3_, F, I, NO_2_, or t-butyl) functional groups. Due to its high positive hydrogen-binding Gibbs free energy (∆G_H_ = 1.04 eV), the pristine carbon nanotube (p-CNT) is unsuitable for the HER. To make it active for the HER, we functionalised it with an aryl group, creating a sp^2^–sp^3^ hybridised interface. When aryl-L groups were added to the CNTs, the ∆G_H_ may be tuned to the optimum value (∆G_H_ = 0). Furthermore, in our research, we investigated whether there is a significant correlation between the curvature of the carbon nanotube and the performance of the HER (it should neither be too large nor too small). Hence, we found the most suitable result for the (6,6) armchair nanotube, with the second best being the (4,4) armchair nanotube. Our results predict highly efficient catalysts for the HER. The charge transfer of the hybridised sp^2^–sp^3^ interface from the aryl functional group to the carbon nanotube altered the charge distribution of the carbon nanotube, enhancing the adsorption of the H-atom on Aryl-L@SWCNT, according to the electronic structure study. In our calculations, aryl-functionalised electro-catalysts were produced, and our results open the door to a new class of low-cost and efficient HER catalysts.

## Data Availability

A Supplementary Information Document has been submitted where raw research data and data supporting reported results can be found. Document can be made open to public.

## Supplementary Materials

The following supporting information can be downloaded at: https://www.mdpi.com/article/10.3390/nano13142122/s1. A separate Supplementary Information document has been submitted with the manuscript. Figures S1–S4: are the graphical representation of Gibbs free energy (ΔG) for (4,4), (6,6), (8,8), and (10,10) armchair SWCNT, respectively; Figure S5: Structural representation of 6,6 armchair SWCNT; Figure S6: Final optimised geometry of Aryl-CCH@SWCNT-4,4 armchair; Figure S7: Final optimised geometry of Aryl-CCH@SWCNT-6,6 armchair; Figure S8: Final optimised geometry of Aryl-CCH@SWCNT-8,8 armchair; Figure S9: Final optimised geometry of Aryl-CCH@SWCNT-10,10 armchair; Figure S10: Influence of H coverage on the Aryl-SWCNT-6,6 armchair. H1, H2, H3, and H4 represent 1 H atom, 2 H atoms, 3 H atoms, and 4 H atoms respectively. Table S1: Bader charge analysis for Aryl-CCH@SWCNT taking 6,6 armchair nanotube. Table S2: Effect of the solvated surface with water solvent (implicit) on six of the newly discovered aryl catalysts.
