# Supplementary material for: Activating Hydrogen Evolution Reaction on Carbon Nanotube via Aryl Functionalisation: The Role of Hybrid sp2–sp3 Interface and Curvature
Source: Nanomaterials (Basel). 2023 Jul 21;13(14):2122. doi: 10.3390/nano13142122 (PMC10385873; doi:10.3390/nano13142122)

Supplementary Information: Activating Hydrogen Evolution Reaction on Carbon Nanotube via Aryl Functionalization: The Role of Hybrid sp^2-sp^3 Interface and Curvature

Muhammad Ahmed[1,2], Gurpreet Kour[1,2], Ziqi Sun[1,2], Aijun Du[1,2,*], and Xin Mao[1,2,*]

1 School of Chemistry and Physics, Faculty of Science, Queensland University of Technology, Gardens Point Campus, Brisbane, QLD-4001, Australia
2 QUT Centre for Materials Science, Queensland University of Technology, Gardens Point Campus, Brisbane, QLD-4001, Australia
* Correspondence: aijun.du@qut.edu.au; Tel: +61 7 3138 6980

Graphical representation of Gibbs Free Energy (ΔG) for 4,4 Armchair

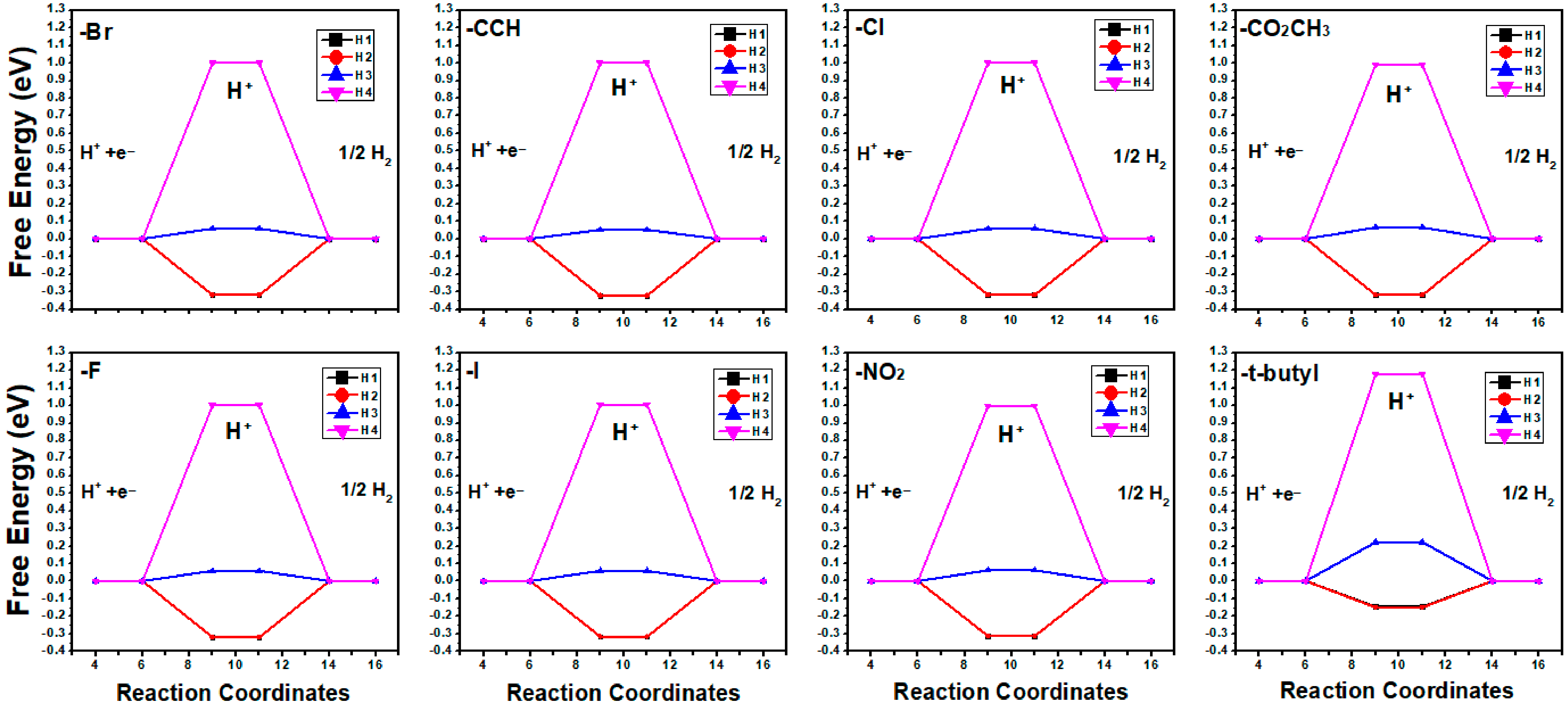

Figure S1. Graphical representation of Gibbs Free Energy (ΔG) for (4,4) armchair SWCNT

Graphical representation of Gibbs Free Energy (ΔG) for 6,6 Armchair

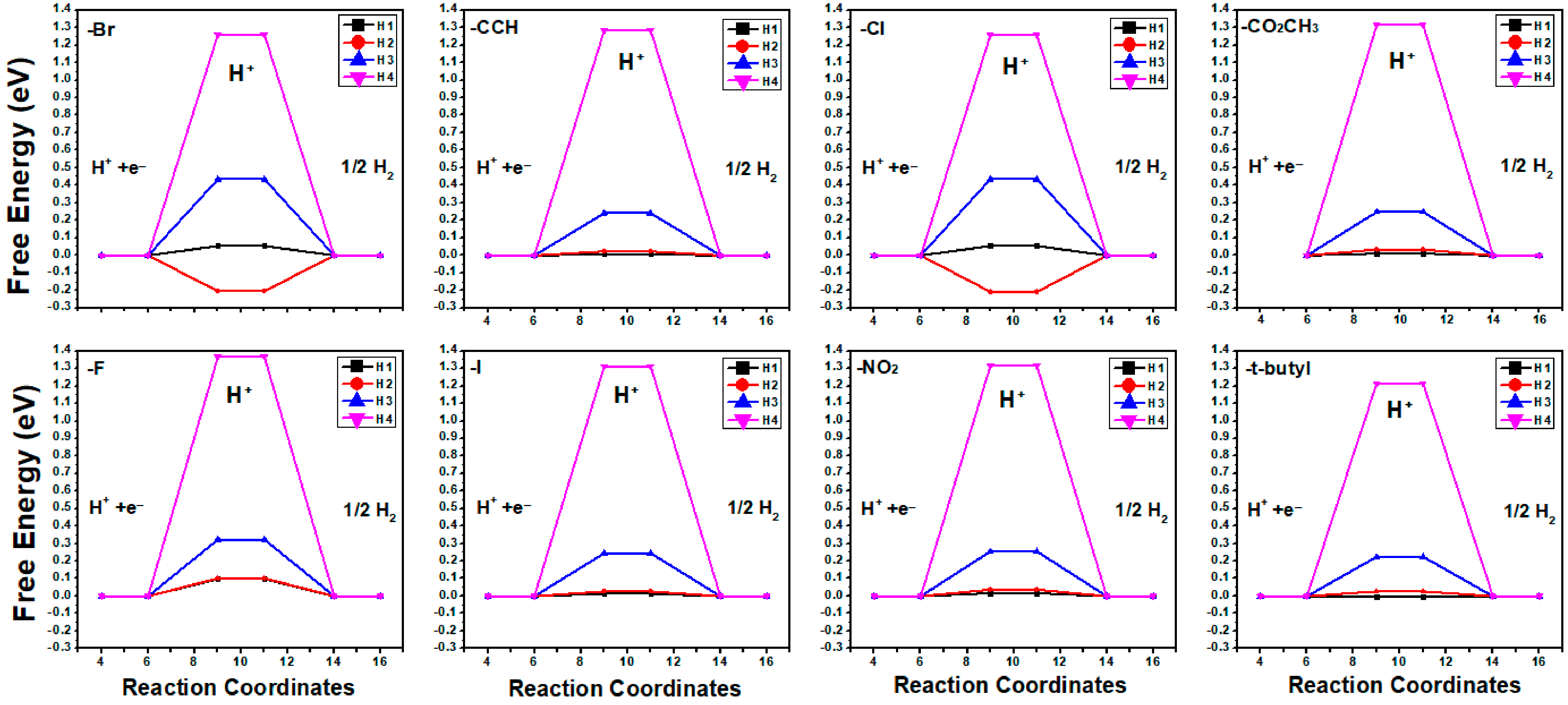

Figure S2. Graphical representation of Gibbs Free Energy (ΔG) for (6,6) armchair SWCNT.

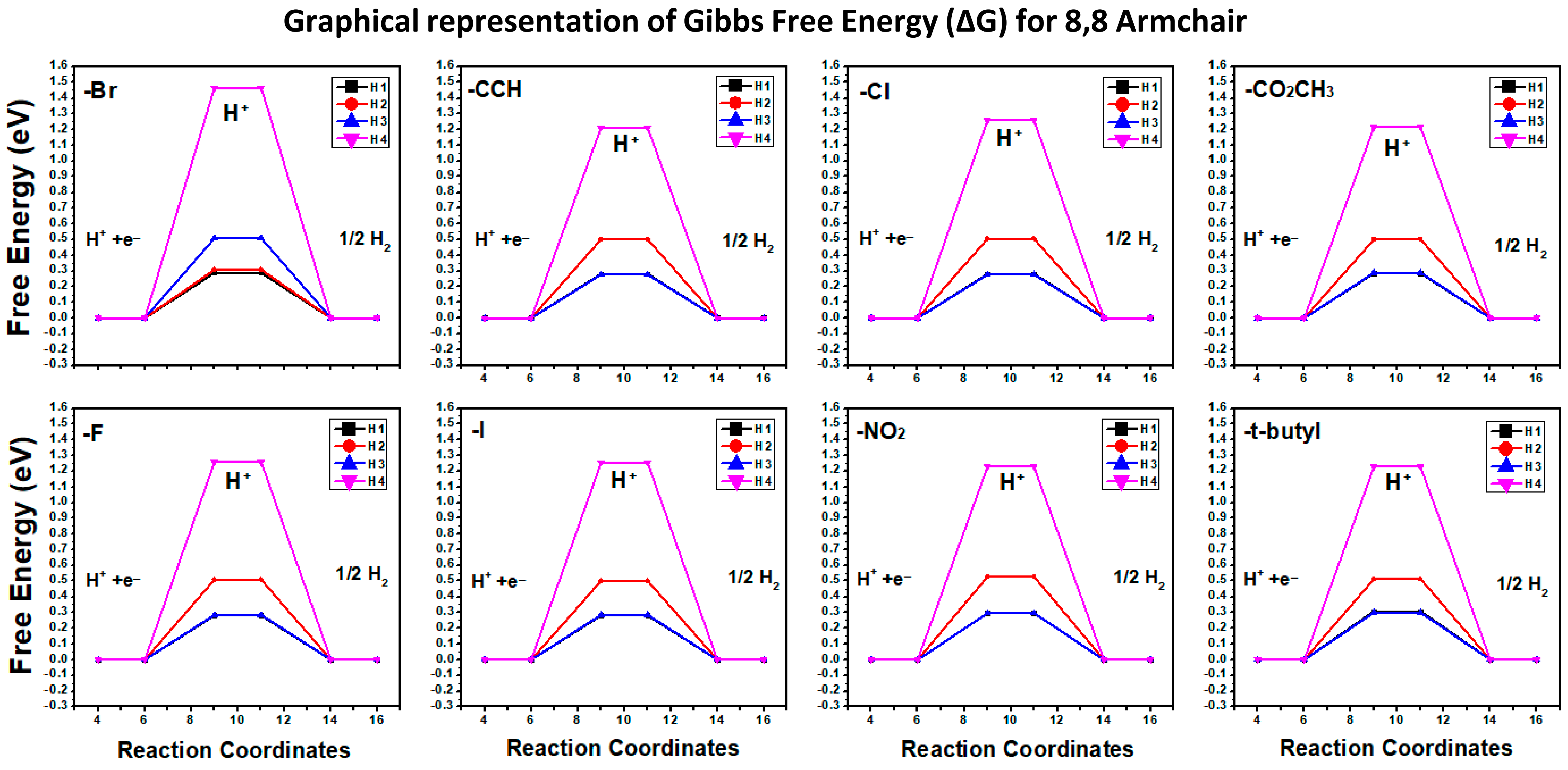

Figure S3. Graphical representation of Gibbs Free Energy (ΔG) for (8,8) armchair SWCNT.

Graphical representation of Gibbs Free Energy (ΔG) for 10,10 Armchair

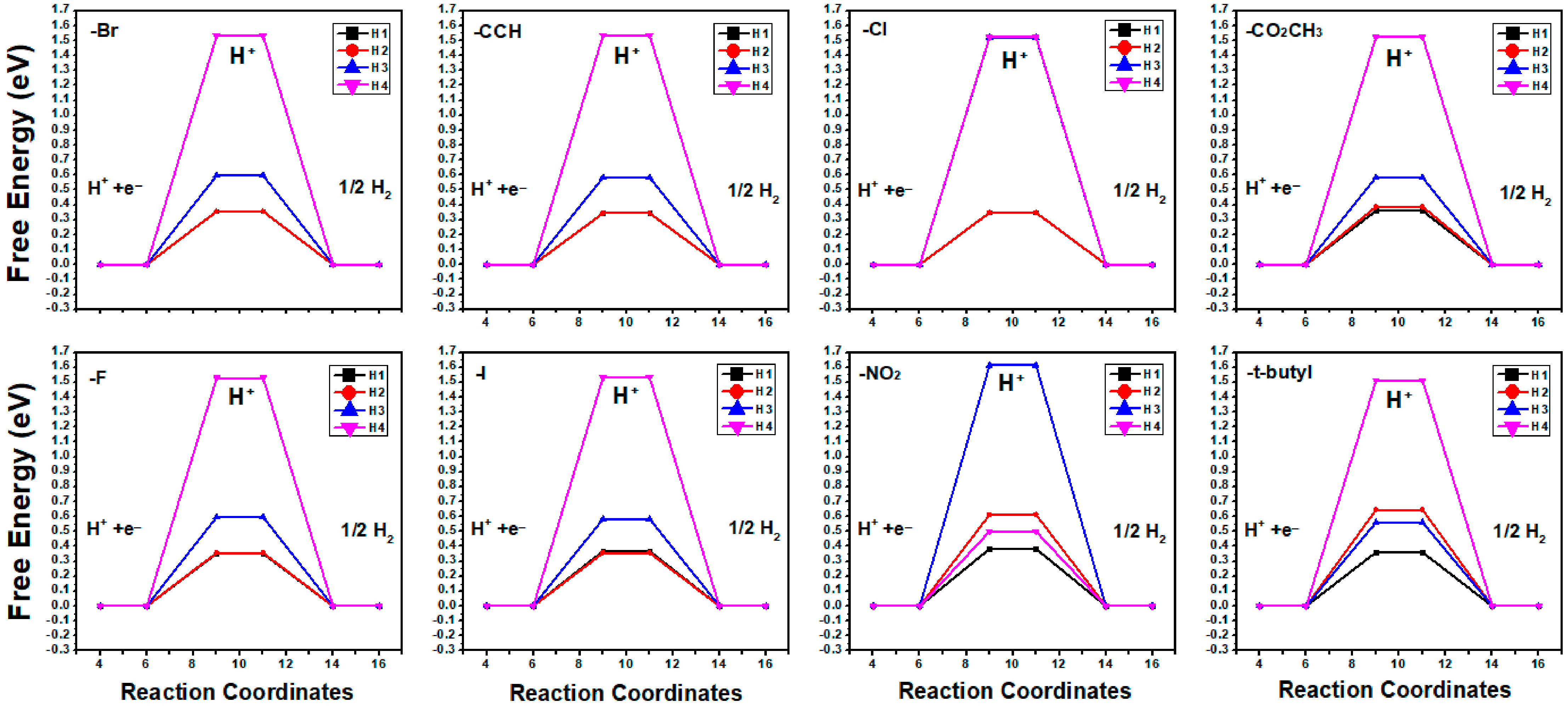

Figure S4. Graphical representation of Gibbs Free Energy (ΔG) for (10,10) armchair SWCNT.

Optimized geometrics for the various intermediates which are formed during the aryl functionalized single walled carbon nanotube

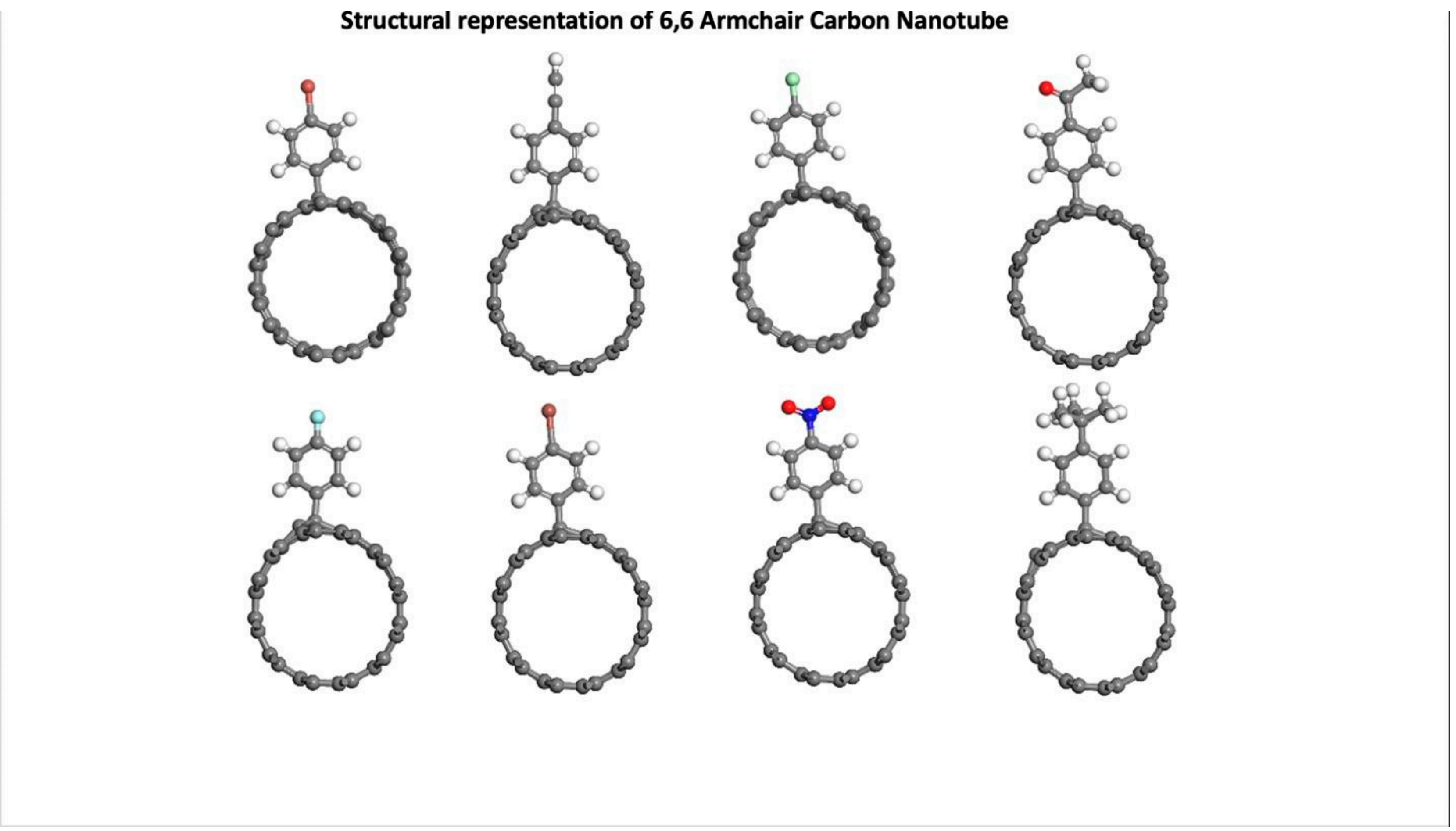

Figure S5. Structural representation of 6,6 armchair SWCNT.

Final optimized geometry of Aryl-CCH@SWCNT-4,4 Armchair

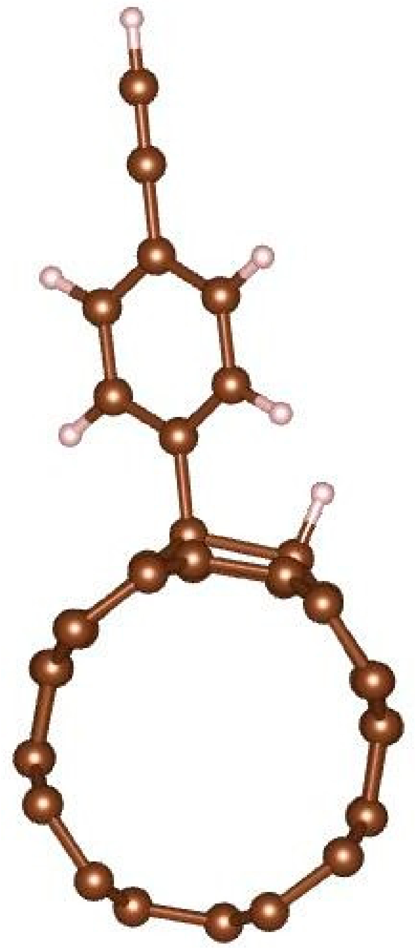

Figure S6. Final optimized geometry of Aryl-CCH@SWCNT-4,4 Armchair.

Final optimized geometry of Aryl-CCH@SWCNT-6,6 Armchair

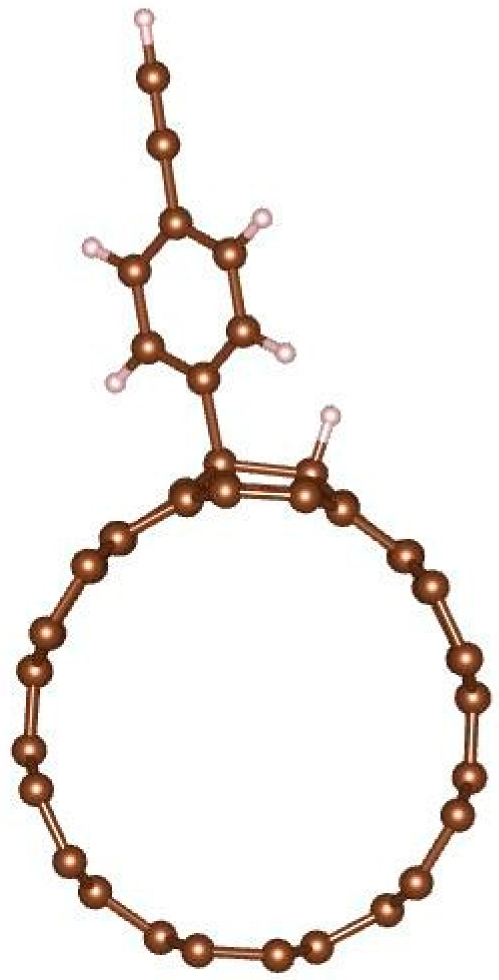

Figure S7. Final optimized geometry of Aryl-CCH@SWCNT-6,6 Armchair.

Final optimized geometry of Aryl-CCH@SWCNT-8,8 Armchair

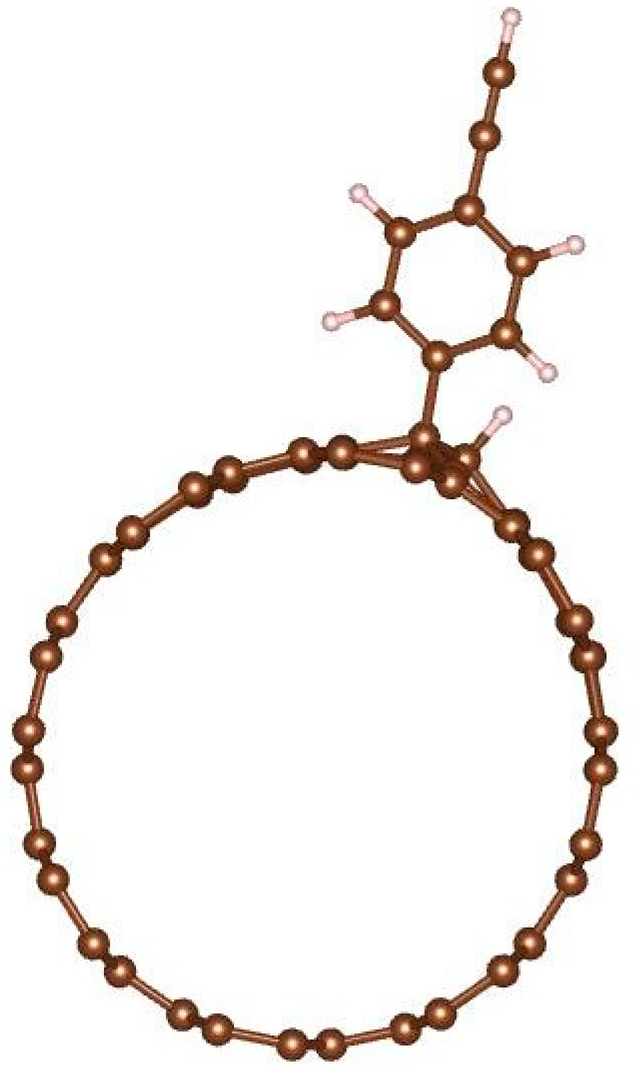

Figure S8. Final optimized geometry of Aryl-CCH@SWCNT-8,8 Armchair.

Final optimized geometry of Aryl-CCH@SWCNT-10,10 Armchair

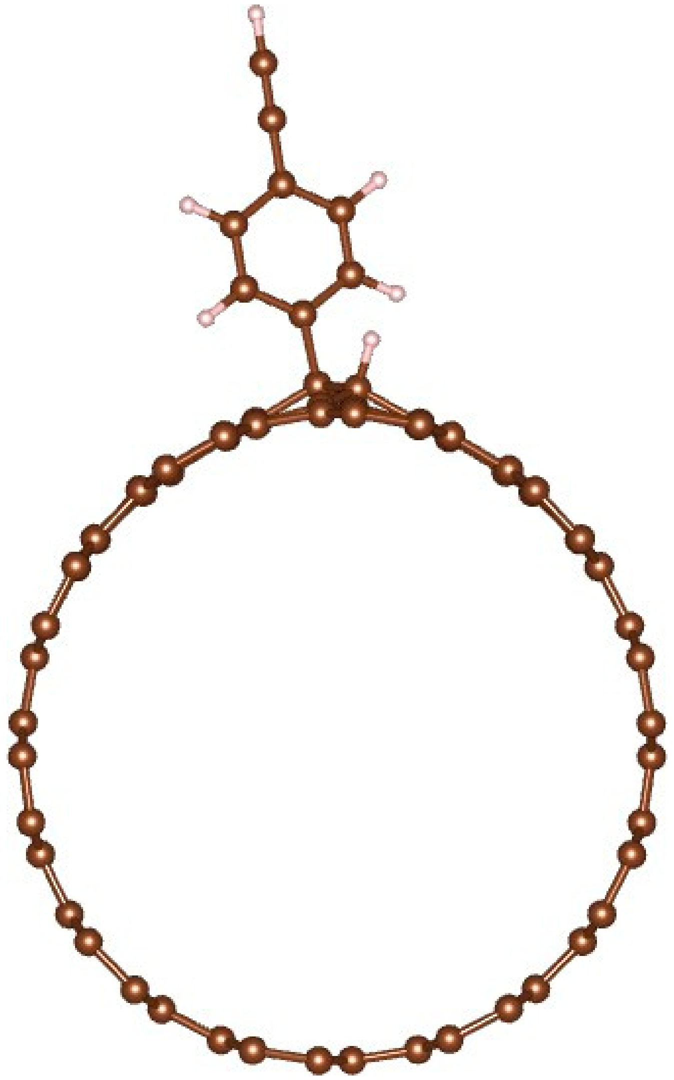

Figure S9. Final optimized geometry of Aryl-CCH@SWCNT-10,10 Armchair.

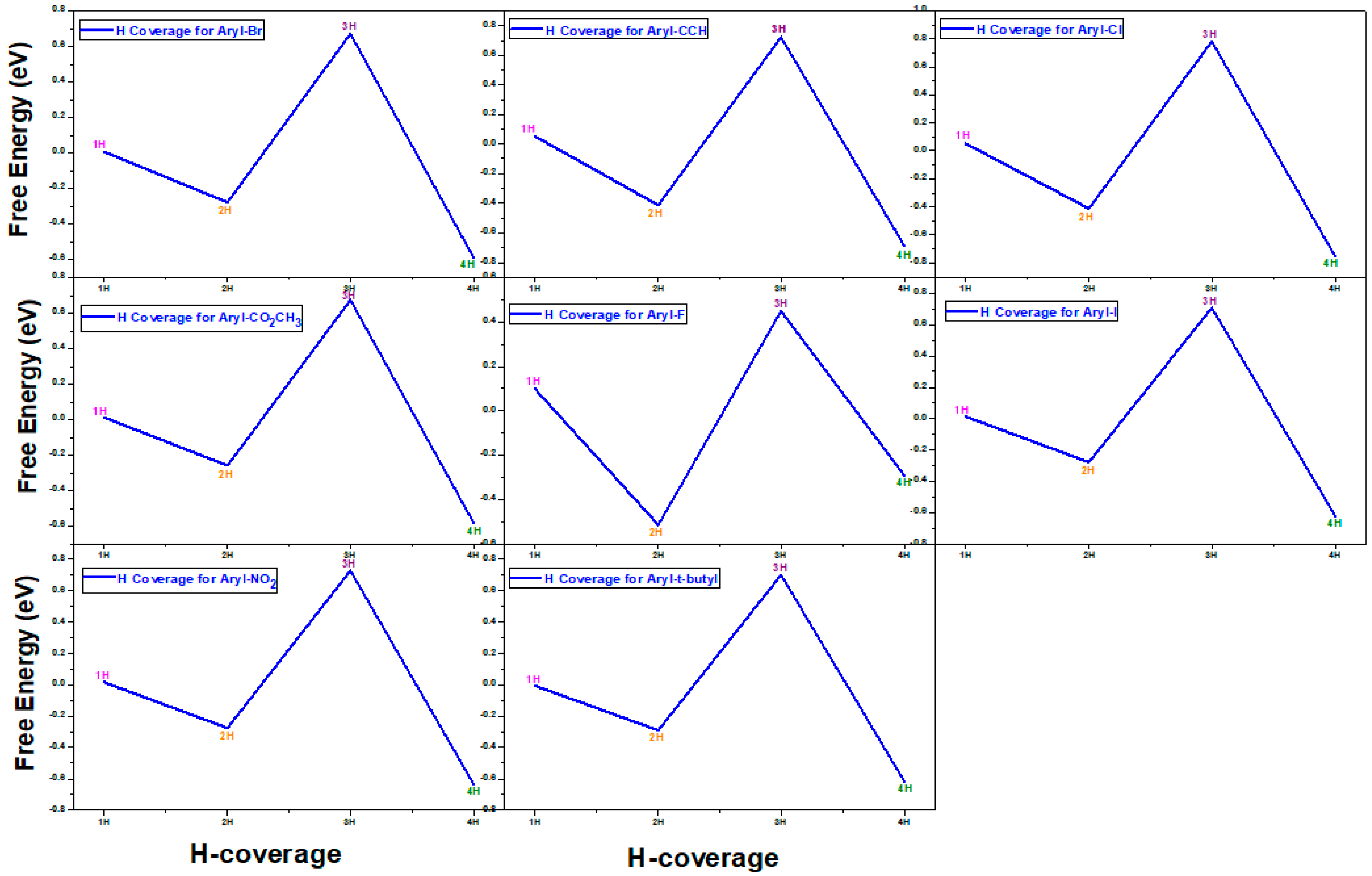

Figure S10. Influence of H coverage on the Aryl-SWCNT-6,6 armchair. H1, H2, H3, and H4 represent 1 H atom, 2 H atoms, 3 H atoms, and 4 H atoms respectively.

Aryl	Charge	Bader charge
**Br**	0.907	-0.092
**CCH**	0.934	**-0.066**
**Cl**	0.92	-0.08
**CO_2CH_3**	0.89	-0.107
**F**	0.90	-0.092
**I**	0.89	-0.105
**NO_2**	0.897	-0.102
**t-butyl**	0.91	-0.09

Table S1. Bader charge analysis for Aryl-CCH@SWCNT taking 6,6 armchair nanotube.

**Aryl Functional Group**	**Without solvation**	**Solvated Surface with Water Solvent (implicit)**
**CCH**	0.005888 eV	**0.011488 eV**
**Cl**	0.053388 eV	**0.047888 eV**
**CO_2CH_3**	0.010988 eV	**-0.044112 eV**
**F**	0.099488 eV	**0.107388 eV**
**NO_2**	0.017188 eV	**0.013888 eV**
**t-butyl**	-0.005812 eV	**-0.072712 eV**

Table S2. Effect of the solvated surface with water solvent (implicit) on six of the newly discovered aryl catalysts.

4,4

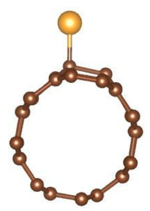

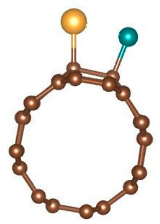

6,6

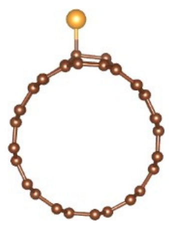

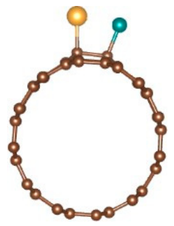

8,8

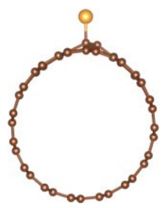

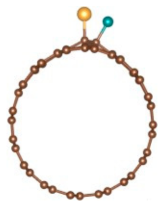

10,10

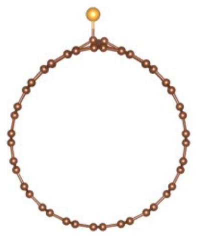

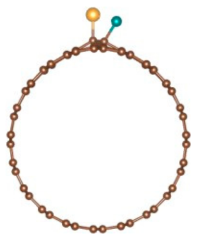

Supplement: Supplementary file 1 [file nanomaterials-13-02122-s001.zip › nanomaterials-2457722-supplementary.pdf]
